# Signal transducer and activator of transcription 3 in myeloid-derived suppressor cells: an opportunity for cancer therapy

**DOI:** 10.18632/oncotarget.8311

**Published:** 2016-03-23

**Authors:** Inès Dufait, Els Van Valckenborgh, Eline Menu, David Escors, Mark De Ridder, Karine Breckpot

**Affiliations:** ^1^ Department of Radiotherapy, Vrije Universiteit, UZ-Brussel, Brussels, Belgium; ^2^ Laboratory of Molecular and Cellular Technology, Vrije Universiteit, UZ-Brussel, Brussels, Belgium; ^3^ Laboratory of Hematology and Immunology, Vrije Universiteit, UZ-Brussel, Brussels, Belgium; ^4^ Immunomodulation Group, Navarrabiomed-Fundaçion, Miguel Servet, IdiSNA, Navarra, Spain

**Keywords:** MDSC, STAT3, T cell, immunotherapy, radiotherapy

## Abstract

Cancer progression is in part determined by interactions between cancer cells and stromal cells in the tumor microenvironment (TME). The identification of cytotoxic tumor-infiltrating lymphocytes has instigated research into immune stimulating cancer therapies. Although a promising direction, immunosuppressive mechanisms exerted at the TME hamper its success. Myeloid-derived suppressor cells (MDSCs) have come to the forefront as stromal cells that orchestrate the immunosuppressive TME. Consequently, this heterogeneous cell population has been the object of investigation. Studies revealed that the transcription factor signal transducer and activator of transcription 3 (STAT3) largely dictates the recruitment, activation and function of MDSCs in the TME. Therefore, this review will focus on the role of this key transcription factor during the MDSC's life cycle and on the therapeutic opportunities it offers.

## INTRODUCTION TO SIGNAL TRANSDUCER AND ACTIVATOR OF TRANSCRIPTION 3

The signal transducer and activator of transcription (STAT) family is comprised of 7 members that are encoded by distinct genes. Because STAT3 is evolutionary the most conserved, it's considered to be a very important member of the STAT family [1]. Similar to its other family members, STAT3 is present in non-stimulated cells in an inactive cytoplasmic form. Activation of STAT3 can be triggered through a multitude of factors among which interleukin-6 (IL-6) like cytokines [2], colony stimulating factors (CSF) and leptin [3], interferon (IFN) as well as IL-2 family members, and growth factors like epidermal growth factor [4]. Depending on the trigger, STAT3 activation occurs through phosphorylation on tyrosine 705 or serine 727. Phosphorylation on tyrosine 705 can be regulated by different tyrosine kinases and by members of the Janus-activated kinases (JAK) [5], whereas phosphorylation of serine 727 can be regulated by protein kinase C, mitogen-activated protein kinases and cyclin-dependent kinase 5 [6]. Phosphorylation of STAT3 results in its dimerization, which enables STAT3 to act as a transcriptional activator of various target genes. Also acetylation of lysine 685 has been described as a mode of STAT3 activation [7] and a way to enhance the stability of STAT3 dimers [6].

**Figure 1:**
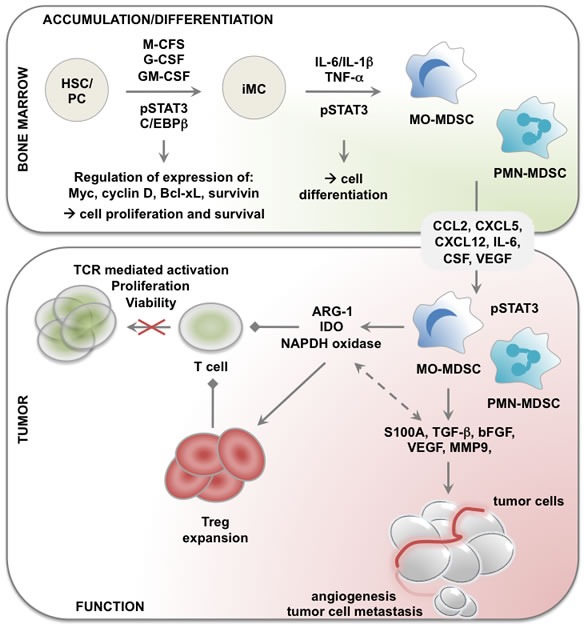
Role of STAT3 in accumulation, differentiation and functional regulation of MDSCs in cancer Cytokines like M-, G- and GM-CSF stimulate myeloid cell development from hematopoietic stem cells (HSCs). Increased production of these cytokines during tumorogenesis interferes with normal myeloid development resulting in the generation of immature myeloid cells. In presence of factors like IL-6, L-1β and TNF−α, these differentiate into MDSCs. Furthermore, cancer cells secrete factors like PGE2 and CXCL12 that help in the recruitment of MDSCs to the TME. Finally, the activation of STAT3 pathway results in the expression of several factors like ARG-1, IDO, TGF-β, ROS etc. These are involved in mediating the tumor promoting function of MDSCs. The arrows “→” indicate “results in; the arrows “← →” indicate interconnectivity and the arrows “→” indicate a suppressive effect. Abbreviations: ARG-1: arginase-1; CXCL12: chemokine C-X-C motif ligand 12; G-CSF: granulocyte-colony stimulating factor; GM-CSF: granulocyte macrophage-CSF; HSC: hematopoietic stem cell; IDO: indoleamine 2,3 deoxygenase; IL: interleukin; iMC: immature myeloid cell; M-CSF: macrophage-CSF; MDSC: myeloid-derived suppressor cell; PC: progenitor cell; PGE2: prostaglandin E2; ROS: reactive oxygen species; STAT3: signal transducer and activator of transcription 3; TGF-β: transforming growth factor-β; TME: tumor microenvironment; TNF-α: tumor necrosis factor-α.

All transcriptional activity requires tight control, which in the case of STAT3 is performed by various negative regulators such as protein inhibitor of activated STAT proteins [8], suppressors of cytokine signaling (SOCS) proteins [9] and protein tyrosine phosphatases [10], [11]. These families of STAT3 regulating proteins interfere with STAT3 binding to DNA, hamper tyrosine kinases and remove phosphates from activated STAT3, respectively. In addition, STAT3 levels can be regulated through ubiquitination-dependent proteosomal degradation [12]. A large body of evidence has shown that STAT3 is constitutively activated in many mouse tumor models [13–16], and more importantly in human cancers including breast, liver, lung, pancreas, prostate, skin, hematological and brain cancers [17–25]. This is explained by the fact that many of the triggers that activate STAT3 are abundantly present in the tumor microenvironment (TME). Moreover, a number of genes induced by STAT3 provide a positive feedback and as such keep the STAT3 pathway continuously activated. Importantly, STAT3 activation occurs in both cancer cells and the many immune cells that infiltrate tumors, among which myeloid-derived suppressor cells (MDSCs) [6], [13], [24–30]. It has been described that STAT3 is one of the factors that allows crosstalk between the different cell types that are part of the TME. Therefore, STAT3 represents an attractive target for modulation. Although activated STAT3 is not only expressed in MDSCs, we will limit the remainder of this review to the role of STAT3 in these myeloid cells (Figure 1), as in contrast to other tumor-infiltrating immune cells like dendritic cells (DCs), macrophages and T cells, MDSCs are abundantly present in most mouse tumor models and human cancers [26], [31]. Moreover, it's becoming increasingly clear that various cancer therapies, such as cancer vaccination, are more effective when MDSCs are depleted [32–38].

## INTRODUCTION ON MYELOID-DERIVED SUPPRESSOR CELLS

Although MDSCs were described in 1970 as natural suppressor cells [39], it took until 2007 for the term MDSCs to get established. Generally the name MDSCs is used to categorize a heterogeneous mix of immature myeloid cells, which can be found in various pathological conditions, including cancer [40]. In healthy individuals, immature myeloid cells, which differentiate into mature macrophages, DCs and granulocytes, are constantly generated in the bone marrow. In cancer bearing subjects, the differentiation of immature myeloid cells is disturbed through the presence of tumor-derived factors that favor immature myeloid cell accumulation and differentiation to MDSCs both at the tumor site and secondary lymphoid organs [41]. Recruitment of MDSCs to the TME is mediated by chemokines such as chemokine C-C motif ligand 2 (CCL2), chemokine C-X-C motif ligand 5 (CXCL5) and CXCL12 [42], [43], as well as other factors such as IL-6, IL-1β, granulocyte-CSF (G-CSF) and vascular endothelial growth factor (VEGF) [44].

In mice, MDSCs are defined as CD11b and Gr-1 expressing cells [45]. Antibodies recognizing the granulocyte-specific marker Gr-1 target an epitope that is shared among the antigens Ly6C and Ly6G, two markers that have been used to divide MDSCs in monocytic (MO, Ly6C^high^Ly6G^low^) and polymorphonuclear (PMN, Ly6C^high^Ly6G^high^) cells [46]. Corresponding populations have been described in cancer patients. In general, human MDSCs are characterized by the expression of CD33, CD11b and the absence of significant levels of lineage markers and HLA-DR [47]. Human MO-MDSCs are further characterized as CD14^+^ but CD15^−^ cells, while PMN-MDSCs are defined as CD14^−^ CD15^+^ [26]. Several other surface markers have been put forward to distinguish MDSC subsets based on their function, among others CD40, CD49 (VLA4), CD80 (B7.1), CD115 (M-CSFR), CD124 (IL4Rα) and CCR2 [27], [48–53]. Although these markers are undoubtedly expressed on MDSCs, it's generally accepted that they do not define specific MDSC subsets [54]. Moreover, the expression of some markers like CD80 can vary considerably depending on the cancer type and MDSC location [55]. Because of this phenotypic heterogeneity, it has frequently been suggested that the suppressive activity of MDSCs is the ultimate defining characteristic [56]. The latter is, in part, dictated by the activation of STAT3 in MDSCs. An unambiguous link between STAT3 and MDSCs is further evidenced by the fact that factors needed to phosporylate STAT3 are also associated with the activation and expansion of MDSCs. These include VEGF, granulocyte macrophage-CSF (GM-CSF), IL-6, basic fibroblast growth factor (bFGF), et cetera. Moreover, factors produced during and following activation of STAT3 are in turn essential for the accumulation and differentiation of MDSCs.

## MYELOID-DERIVED SUPPRESSOR CELLS: A LIMITATION TO CANCER IMMUNOTHERAPY

Cancer immunotherapy is based on the evidence that the immune system can discriminate between cancer cells and healthy cells, since the former express tumor antigens [57]. Based on this premise, cancer immunologists believe that it's possible to stimulate tumor-specific cytotoxic T lymphocytes (CTLs) to reject and eliminate cancer cells. Several strategies have been explored of which therapeutic cancer vaccination [58–63], adoptive T-cell transfer [64], [65] and more recently blockade of inhibitory receptors such as programmed cell death-1 (PD-1) and cytotoxic T lymphocyte-associated antigen-4 (CTLA-4) [66–72] have shown promising results. Consequently, cancer immunotherapy has become a fourth treatment strategy within the clinician's toolbox. Despite long-term tumor control in subsets of patients, it is frequently observed that in most cases where tumor-specific CTLs can be detected, they are unable to cause tumor regression. This finding hints that in these patients, mechanisms other than inhibitory immune checkpoint triggering override the function of tumor-specific T cells. Cancer cells have adapted several immune-avoiding mechanisms, for example the loss of tumor antigens or MHC I expression [73], and the recruitment of suppressive immune cells [74]. It has become increasingly clear that immunotherapy also has to interfere with the function of suppressive immune cells at the TME. Defining which suppressive immune cell types should be targeted and how, is a challenging task. In this regard, MDSCs have come to the forefront as a target population, because they are prevalent in most cancer types, both murine and human, and because they exploit a plethora of mechanisms to directly or indirectly abrogate anti-tumor immunity [75]. However, the heterogeneity of MDSCs and the diversity of inhibitory mechanisms they employ have faced us with the challenge of finding a “one fits all” strategy to deplete and/or functionally modulate them. Fortunately, the cell's behavior is in large dictated by transcriptional programs. In the case of MDSCs, it has been suggested that the transcription factor STAT3 is a main regulator [75]. This is further highlighted by the observation that STAT3 expressed by MDSCs is implicated in their accumulation, differentiation and functionality (Figure 1).

## SIGNAL TRANSDUCER AND ACTIVATOR OF TRANSCRIPTION 3 PLAYS A ROLE IN THE ACCUMULATION AND DIFFERENTIATION OF MYELOID-DERIVED SUPPRESSOR CELLS

Cancer-derived factors that drive the generation of MDSCs in the bone marrow include G-CSF and GM-CSF, various interleukins like IL-6 and IL-1β, prostaglandin E2 (PGE2), tumor necrosis factor-α and VEGF [42]. Many of these activate the STAT3 pathway, so it's no surprise that STAT3 signaling has been implicated in the stimulation of myeloid cell differentiation into MDSCs. STAT3 interacts with CCAAT-enhancer-binding protein β (C/EBPβ). This transcription factor has a key role in myeloid development, as C/EBPβ-deficient bone marrow cells lose the ability to differentiate into functional MDSCs [76].

Furthermore, a correlation between C/EBPβ and accumulation of CD11b^+^ Gr-1^+^ cells in response to G-CSF was reported [77], [78]. This observation and the finding that STAT3 deficiency makes myeloid progenitors refractory to growth stimulation by G-CSF [79], suggests that STAT3 and C/EBPβ are inextricably linked in MDSC generation. This is further supported by the observation that STAT3 prolongs the binding of C/EBPβ on the myc promoter [76]. Besides myc, other cell survival and cell cycle regulating proteins like Bcl-xL, survivin, Mcl-1 and cyclin D1 are upregulated by STAT3 [6], [31], [80]. STAT3 was further linked to proteins like S100A [81] and protein kinase C βII [82], which inhibit DC differentiation from myeloid progenitor cells and thereby promote MDSC accumulation.

The studies described above clearly point towards a role for STAT3 in MDSC expansion and differentiation. In addition, there are multiple other possible mechanisms in which STAT3 can influence MDSCs. MicroRNAs (miRs) have been proven to be crucial in the regulation of myeloid cell maturation, activation, proliferation and differentiation [83], [84]. MiR155 is generally thought to be immune stimulatory by controlling lymphocyte differentiation and function [85], [86], but has recently been shown to promote the expansion of functional MDSCs [87]. In addition, miR155 has been found, together with miR21, to be the most upregulated miR during the induction of MDSCs from bone marrow. Genetic ablation of miR155 renders mice resistant to chemical-induced tumors suggesting that it also exerts its functions in immunosuppression and tumor promotion. When miR155 is absent, the suppressive functions of MDSCs are impaired through SOCS1 and an increase in SH2 (Src homology 2)-containing inositol phosphatase-1 (SHIP-1) [88]. In accordance, the study by Li et al showed a synergistic effect of miR155 on MDSCs induction via targeting of SHIP-1, phosphatase and tensin homolog, subsequently leading to STAT3 activation [87]. Moreover, miR155 can regulate inflammatory cytokine production through targeting of C/EBPβ in macrophages and MDSCs. It has been shown that the expression of C/EBPβ is inversely correlated with the amount of miR155 [89–91]. Furthermore, depletion of C/EBPβ can potentiate the transforming growth factor-β (TGF-β) response and contributes to cancer progression due to TGF-β induced endothelial-to-mesenchymal transition (EMT) [92].

The link between STAT3 activation and miR155 has been reported earlier in the context of experimental autoimmune uveitis [93] and laryngeal squamous carcinoma [94]. Moreover, it appears that these different mechanisms are interconnected. A novel axis between, S100A4, miR155, SOCS1 and matrix metalloproteinase (MMP) was unveiled in hepatocellular carcinoma. S100A4 upregulates miR155, which suppresses SOCS1, activates STAT3 signaling and in turn enhances MMP9, promoting tumor invasiveness [95]. Furthermore, the increase in miR155 correlates with upregulation of oncogenes, such as cyclin D1 and c-myc [96]. Interfering with any aspect of this axis could present a useful therapeutic approach for controlling proliferation and metastasis.

Activation and expansion of MDSCs can also be mediated by the release of tumor-derived soluble factors but recent reports have shown a role for microvesicles, namely exosomes, in MDSC biology [97], [98]. Exosomes are endosome-derived organelles with sizes of 50 to 150 nm, which are actively secreted through an exocytosis pathway to serve as mediators of intracellular crosstalk [99]. They can contain different cargo including miRs, proteins and lipids, which are then delivered to the receptor cell. Their story can be deemed identical to the miR story: exosomes were initially described to be immune stimulatory but recent research contradicts this statement, showing a role of exosomes in both inducing MDSCs [100], and inhibiting T-cell function or DC differentiation [101]. However, discrepancies in this field still exist, as the exact effects of exosomes on MDSCs are not fully elucidated yet. Chalmin et al describe that heat shock protein 72 (HSP72) expressing exosomes derived from different solid tumor cell lines, account for MDSC activation through triggering of STAT3 in a Toll-like receptor 2 (TLR2)/myeloid differentiation protein 88 (MyD88)-dependent manner through an autocrine IL-6 production, whereas tumor-derived soluble factors are responsible for MDSC expansion [102]. In the model by Xiang et al exosomes derived from the supernatants of cultured tumor cells (C-exo) induced both MDSC activation and expansion. It was suggested that this discrepancy could be attributed to the presence of PGE2 in the C-exo, while no PGE2 was detected in the exosomes used by Chalmin et al [100], [102].

Moreover, we found in a multiple myeloma mouse model that exosomes derived from bone marrow stromal cells or multiple myeloma cells themselves could activate both the STAT1 and STAT3 pathway leading to expansion and increased suppressive activity of the MDSCs [103]. This activation was independent of GM-CSF or HSP72 (unpublished data). Of note, there is a lot of controversy about the different techniques used to isolate exosomes [104]. While ultracentrifugation was long time the norm, it is nowadays acknowledged that contaminating lipids and proteins remain. These could be at the basis of contradictory data. Therefore studies concerning exosomal effects should be regarded with caution.

## SIGNAL TRANSDUCER AND ACTIVATOR OF TRANSCRIPTION 3 AND ITS ROLE IN THE TUMOR PROMOTING ACTIVITY OF MYELOID-DERIVED SUPPRESSOR CELLS

Several mechanisms are employed by MDSCs to promote tumor growth, including suppression of anti-tumor immune responses, stimulation of angiogenesis as well as tumor cell metastasis. These activities have been linked to activation of STAT3 in the MDSCs.

### 1) Inhibition of anti-tumor immune responses

Immune suppression is the most important biological characteristic of MDSCs. To that end, MDSCs deplete nutrients required by T cells for their clonal expansion, generate oxidative stress leading to reactive oxygen species (ROS) production, activate and expand regulatory T cells (Tregs), and finally inhibit T-cell trafficking [31]. Several mechanisms that are at the basis of these MDSC activities have been linked to activation of STAT3.

Expression of arginase-1 (ARG-1) is under the control of STAT3 and results in consumption of L-arginine and L-cysteine from the TME [26], [105–109]. Depletion of these amino acids results in downregulation of the CD3ζ-chain in the TCR complex and growth arrest of antigen-activated T cells [110], [111]. Moreover, Serafini et al linked the expression of ARG-1 to expansion of Tregs by MDSCs in a B-cell lymphoma model [112]. In this particular model TGF-β produced by the MDSCs had no effect on Tregs. Nonetheless, TGF-β has been linked to T-cell suppression [113], Treg expansion [25], [114] and initiation of EMT [92]. Importantly, a link between TGF-β and STAT3 was proposed based on the presence of two STAT3 binding sites in the TGF-β promoter [115]. Moreover, it was shown that TGF-β production was reduced after myeloid-specific STAT3 knock down [25]. This reduction in TGF-β was correlated to a reduction in Treg numbers. Another enzyme that is under the control of STAT3 and that depletes an essential T-cell nutrient is indoleamine 2,3 deoxygenase (IDO) [116]. STAT3-induced upregulation of IDO can be mediated by three molecular mechanisms, including binding of STAT3 to the promotor region of the IDO gene [117], and an indirect regulation of IDO expression via activation of C/EBPβ [118], [119] or non-canonical activation of NF-κB [120], [121]. Importantly, in human breast-cancer derived MDSCs non-canonical activation of NF-κB in an IL-6/STAT3-dependent fashion has been proposed as the predominant mechanism [122]. IDO depletes tryptophan thereby generating the toxic metabolite kynurenine. The mode of action of IDO is similar to that of ARG-1, suppression of TCR-mediated effector T-cell activation, growth arrest, induction of effector T-cell apoptosis and expansion of Tregs [123], [124].

Besides amino acid deprivation, STAT3 phosphorylation in MDSCs has also been linked to the activation of two subunits of NADPH oxidase (NOX2), namely P47^phox^ and gp91^phox^, leading to an increased generation of intracellular ROS, another mechanism that dampens anti-tumor immunity [6], [28], [125]. It was postulated that S100A8/A9 heterodimers assist in the formation of the NADPH oxidase complex [79]. Moreover, ARG-1 can also contribute to ROS production [126], [127]. Importantly, ROS play a role in the suppression of antigen-specific T cells [128–130] and has been shown to induce T-cell apoptosis [131], much in the same way as ARG-1. The leading hypothesis states that ARG-1 is mostly expressed by PMN-MDSCs due to activation of STAT3, while MO-MDSCs mostly express inducible nitric oxide synthase (iNOS) through the activation of STAT1 and STAT6 [132], [132]. Recently, evidence contradicting this view has emerged. It was shown both *in vitro* and *in vivo* that inhibitors of iNOS suppressed VEGF release, induced STAT3 activation and ROS production [133]. Additionally, in human cells both the promotor of ARG-1 and iNOS have STAT3-binding elements, suggesting that STAT3 is not exclusively linked to ARG-1[105]. Moreover, activation of NF-κB as a result of STAT3 phosphorylation has been implicated in the regulation of iNOS expression [134]. As this study was performed on macrophages, more in depth research is needed to elucidate the molecular mechanisms that regulate the STAT3/iNOS pathway in MDSCs. Nonetheless, the studies described above demonstrate a central role for STAT3 in the active quenching of anti-tumor immunity by MDSCs.

### 2) Promotion of tumor cell dissemination

Immune suppression is not the only way in which MDSCs support tumor growth. They also promote tumor progression by enhancing blood vessel development, tumor cell invasion and metastasis. Angiogenesis has been linked to enhanced production of VEGF and bFGF by MDSCs. These angiogenic factors are under the control of STAT3 [135]. Moreover, STAT3 driven proteases like MMP9 and TGF-β have also been linked to angiogenesis [43]. In this regard MMP9 was shown to enhance the bioavailability of VEGF and as such support vascular stability [136]. In addition to the role in vasculogenesis, MMP also play a role in promoting tumor cell metastasis. Furthermore, MDSCs expressing active STAT3 have been implicated in the formation of pre-metastatic niches [137], [138]. These cells condition organs by creating an immunosuppressive environment that allows growth of metastatic tumor cells [139–141]. Herein, STAT3 regulated factors like bFGF, interleukins, MMP9 and S100A proteins play a role [139], [142].

### 3) Bidirectional link between tumor cell dissemination and immunity

It was recently shown in a mouse model that CD8^+^ T cells could counteract the formation of pre-metastatic niches by MDSCs by inducing MDSC apoptosis. However, activation of STAT3 compromises the ability of T cells to kill MDSCs [137], [138]. This was linked to lower granzyme B expression by CD8^+^ T cells and resistance of MDSCs to T-cell killing. Importantly, these mouse data are supported by data obtained in melanoma patients. Zhang et al showed a positive correlation between STAT3 activation and myeloid cell accumulation, increased IL-10, IL-6 and VEGF, while they observed an inverse correlation between STAT3 activation and CD8^+^ T cell numbers as well as the expression of granzyme B by T cells in melanoma draining lymph nodes [143].

The studies described above underline the role of STAT3 as a master regulator of the MDSC's tumor promoting activity.

## SIGNAL TRANSDUCER AND ACTIVATOR OF TRANSCRIPTION 3 AND ITS ROLE IN RADIATION RESPONSE

STAT3 also plays a pivotal role in resistance to radiotherapy. Radiotherapy, which is currently used in cancer patients as a standard treatment, next to chemotherapy and surgery, still has certain hurdles to overcome, among which toxicity and (acquired) radiotherapy resistance. A considerable part of primary tumors are (partly) resistant to radiotherapy. A major goal in the field of radiobiology is to re-sensitize these tumors to radiation therapy. The first evidence on a role for STAT3 in radiotherapy resistance originated from a study by Otero et al in 2006 where radiation-induced apoptosis resistant peritoneal B-1 B cell subsets were used. B-1 cells possessed constitutively active STAT3. The radioresistance of B-1 cells could be conferred to radiosensitive B-2 cells by crosslinking in the presence of IL-6. Moreover, the B-1 cells became susceptible to irradiation by knocking out STAT3 [144]. Similarities exist for human cells as it was shown that downregulation of STAT3 enhanced the radiotherapy sensitivity of laryngeal squamous cell carcinoma xenografts. Furthermore, a positive correlation between the expression of STAT3 and Bcl-2 was uncovered [145]. This was further confirmed when it was shown that radiation itself induces phosphorylation of JAK2/STAT3 and increases the levels of Bcl-2 and Bcl-XL [146]. STAT3 affects various biochemical processes; therefore it's very likely that it serves as a modulator of radioresponses in more than one way. We will discuss the interaction of STAT3 and hypoxia-inducible factor (HIF) as the hypoxic environment of the tumor is considered to be the main cause of clinical radiotherapy resistance [143]. In renal cell carcinoma, it has been shown that hypoxia activates STAT3, which consequently binds to the HIF-1α promotor and contributes to the stability and synthesis of the HIF-1α protein [147]. Inhibitors of STAT3 efficiently radiosensitized esophageal-, head and neck squamous cell carcinoma and prostate cancer cells and inhibited both hypoxia/radiation-induced activation of STAT3 and upregulation of HIF-1α and VEGF expression [148–150]. Further *in vitro* and *in vivo* data using a large spectrum of human tumors also convincingly show that JAK/STAT signaling is important in mediating resistance to radiation therapy. This is reviewed elsewhere [151]. Despite this compelling evidence, only the effect of STAT3 in the tumor cells is studied, while immune cells have been largely disregarded. However, there is evidence that downregulation of STAT3 in cancer cells impacts on the number of MO-MDSCs, while influencing the activity of PMN-MDSCs [1]. Moreover, it's increasingly clear that immune cells play an important role in the radiation response, as reviewed elsewhere [152]. The role of MDSCs in radiation response has not been fully elucidated, but evidence has emerged that the response of myeloid cells to radiation is model- and dose- dependent and can be both pro- and anti-tumoral [153]. This raises the question whether the STAT3 status of both tumor and immune cells is important in the general radiation response?

## TARGETING SIGNAL TRANSDUCER AND ACTIVATOR OF TRANSCRIPTION 3 AS A STRATEGY TO MANIPULATE MYELOID-DERIVED SUPPRESSOR CELLS

As mentioned, MDSCs have come to the forefront as a target in cancer (immune)therapy because of several reasons. Firstly, MDSCs are abundantly present in most cancer patients, irrespective of the cancer type [26]. Secondly, the presence of MDSCs correlates with cancer stage and metastatic disease [154]. Thirdly, MDSCs accelerate tumor progression by inhibiting anti-tumor immune responses, stimulating angiogenesis, tumor cell invasion and metastasis [26], [31].

Throughout this review, we showed that STAT3 is implicated in the accumulation, differentiation and function of MDSCs. Consequently, several research teams have evaluated STAT3 targeting drugs as a means to interfere with these processes and as such put a brake on tumor progression [35], [75]. A gene therapy approach has been investigated in a transgenic mouse model that spontaneously develops medulloblastoma tumors. Herein the myeloid cell LysM promotor was driving the expression of Cre recombinase to conditionally delete STAT3 using the Cre/LoxP system. STAT3 removal resulted in a reduction of PMN-MDSCs in the tumor and an increased effector T cell/Treg ratio. However, no changes in tumor incidence were reported [25].

Next to the genetic approach a list of drugs has been investigated to avoid phosphorylation of STAT3. This list includes curcumin derivatives and other JAK2/STAT3 inhibitors including AZD1480 [30], [155–158], Icariin flavone and its derivative 3,5,7-trihydroxy-4′-emthoxy-8-(3-hydroxy-3-methylbutyl)-flavone [159], tyrosine kinase inhibitors such as sunitinib [160–163], VEGF inhibiting molecules such as VEGF-trap (a VEGF receptor fused to the Fc part of human IgG1) [164], [165] and anti-VEGF antibodies (bevacizumab) [166], [167], monoclonal antibodies specific for IL-6 [168], molecules like bardoxolone methyl (CDDO-Me) [33], [169–171] and chemotherapeutics such as docetaxel [155–158] (Table 1).

**Table 1:** Effect of (in)direct targeting of STAT3 on myeloid-derived suppressor cells

Agent	Mechanism of action	Study setting	Tumor model	Study finding	Ref
AZD1480	JAK2/STAT3 inhibitor	Preclinical (*in vivo*)	Melanoma model	Decreased levels of MDSCs, but higher suppressive activity on a per cell basis	30
Curcumin derivatives	JAK2/STAT3 inhibitor	Preclinical (*in vivo*) Patient study (blood samples)	Gastric-colon carcinoma Lung cancer	Inhibits accumulation and induces differentiation of MDSCs Decreased numbers of MDSCs, while increasing mature myeloid cells in peripheral blood	156-157
Icariin flavone and its derivatives	Inhibit STAT3 signaling and expression of S100A8 and S100A9	Preclinical (*in vivo*)	Breast carcinoma	Downregulates MDSC numbers	159
Sunitinib	Receptor tyrosine kinase inhibitor	Preclinical (*in vivo*) Preclinical (*in vivo*) Patient study (blood samples)	Renal-breast-colon carcinoma Breast carcinoma Metastatic renal carcinoma	Eliminates MDSCs. Still debate about the location of depletion. Improves T_H_1 function and lowers T_reg_	160-163
Avastin	Anti-VEGF antibody	Xenografts	Renal cell carcinoma	Reduces the number of circulating myeloid cells	166
Bevacizumab	Anti-VEGF antibody	Patient study (blood samples)	Renal cell carcinoma	No effects on MDSCs in peripheral blood	167
Monoclonal antibodies (specific for IL-6)	Anti IL-6 receptor antibody	Preclinical (*in vivo*)	Skin squamous cell carcinoma	Downregulates accumulation of MO-MDSCs	168
Bardoxolone methyl (CDDO-Me)	Inhibits JAK1 activity	Preclinical (*in vivo*) Patient study (blood samples)	Colon-lung carcinoma Lymphoma model Renal -soft tissue carcinoma	Abrogated immune suppressive activity of MDSCs	33, 171
Docetaxel	Inhibition of pSTAT3	Preclinical (*in vivo*)	Breast carcinoma	Decreased MDSC numbers in the spleen	172
CpG-siSTAT3	TLR9-targeted STAT3 silencing	Preclinical (*in vivo*) Patient study (blood samples-tumor specimens)	Melanoma model Leukemia Prostate carcinoma	Abrogates immunosuppressive activity of MDSCs Induces potent innate anti-tumor responses	178-180
Conditional STAT3 gene disruption	Cre/LoxP system under LysM promoter	Preclinical (*in vivo*)	Medulloblastoma	A significant reduction in the abundance of PMN-MDSCs and Tregs was observed within tumors No effect on tumor incidence in mice	25

Curcumin and its derivatives are naturally occurring phenols that are used for their anti-oxidant and anti-inflammatory activities. Furthermore, these have been used to selectively inhibit the JAK2/STAT3 pathway [155–158]. Administration of Cucurbitacin B (CuB) to lung cancer patients was shown to decrease the numbers of bona fide MDSCs (Lin^−^ HLA-DR^−^ CD33^+^), while it increased the numbers of mature Lin- HLA-DR^+^ CD33^+^ myeloid cells in peripheral blood. Moreover, it was shown *in vitro* that CuB induced DC differentiation and increased the sensitivity of tumor cells to antigen (p53)-specific T cells [156]. Also other JAK2/STAT3 inhibitors have been tested, including AZD1480 [30], which resulted in low levels of MDSCs in tumor bearing AZD1480 treated mice. However, AZD1480 treatment did not abrogate the ability of the remaining MDSCs to suppress T cells. Moreover, when evaluated on a per cell basis, it was shown that the suppressive activity of the MDSCs was higher after treatment with AZD1480. Similar to JAK2/STAT3 inhibitors, flavanoids like Icariin and its derivative were reported to downregulate MDSC numbers [159]. These natural compounds were shown to inhibit STAT3 signaling and expression of S100A8 and S100A9, resulting in differentiation of immature myeloid cells to mature cells. Sunitinib is a small-molecule multikinase inhibitor that targets among others the VEGF receptor, platelet-derived growth factor receptor and c-kit, and as such hampers the phosphorylation of STAT3. Ko et al [160], showed that sunitinib efficiently eliminates peripheral MDSCs, whereas it did not reduce MDSCs in tumors. This was linked to high levels of GM-CSF in the tumor and STAT5 signaling in MDSCs. Nonetheless, other studies show that MDSC depletion by sunitinib is irrespective of the location [162]. Importantly, treatment of metastatic renal cell cancer patients with sunitinib reduced the level of MDSCs in peripheral blood by half and was associated with improved T_H_1 function (reduced IL-4 and higher IFN-γ) and lower Treg numbers [161], [163]. Although sunitinib, which affects downstream VEGF receptor signaling and as such STAT3 activation, was shown to modulate MDSC levels, other strategies that impact on VEGF receptor signaling, such as VEGF-trap [164], [165] and anti-VEGF antibodies demonstrated no effect on MDSC levels in peripheral blood of cancer patients [167]. This is an unexpected finding, since the link between VEGF and MDSC accumulation is longstanding and as it was shown that anti-VEGF antibodies successfully reduce MDSC numbers in mice [166]. Besides antibodies to capture VEGF and as such inhibit STAT3 activation upon VEGF receptor interaction, researchers have developed monoclonal antagonistic antibodies specific for the IL-6 receptor, as it's triggering is directly linked to STAT3 activation and MDSCs. These anti-IL-6 receptor antibodies neutralize the effects of tumor-derived IL-6 and suppress expansion of cancer-associated MDSCs [168]. Another molecule that was shown to inhibit STAT3 activation in MDSCs, at least when used at high concentrations (1-5 μM), is CDDO-Me. Treatment with this synthetic triterpentoid (a methyl ester of 2-cyano-3,12-dioxooleana-1,9 (11)-dien-28-oic acid) resulted in reduced production of ROS, improved T-cell function and more importantly reduced tumor growth [33], [169–171]. Finally, the chemotherapeutic agent, docetaxel was evaluated for its direct effect on MDSCs. Low-dose treatment polarized MDSCs toward an M1-like phenotype, as deduced from the expression of CCR7, MHC II, CD11c and CD68, and reduced the number of MDSCs in the spleen. Administration of docetaxel also resulted in increased CTL responses and reduced tumor growth. These effects were partially attributed to inhibition of STAT3 activation [172]. The studies above show the potential of targeting STAT3 in MDSCs as an anti-cancer strategy. At the same time these studies demonstrate that although the aforementioned drugs act on STAT3 activation their mode of action can differ from MDSC depletion over maturation to functional modulation. Moreover, treatment of cancer bearing subjects with only these drugs was shown to be insufficient to provide a cure. As MDSCs represent a confounding factor for anti-tumor immunity and as it was found that MDSC depletion improves the outcome of cancer vaccines [32–34], [34–38], it's not surprising that drugs such as CDDMO-Me [33] and sunitinib [37], [38], [173] have been evaluated in combination with cancer vaccination. In these studies the combination therapy showed improved curative potential when compared to either component alone. However instead of combining therapies, it would be more elegant if one drug could lead to activation of tumor-specific CTLs, while modulating MDSCs. Importantly, various studies have demonstrated that MDSCs can be reverted into stimulatory APCs under the influence of cytokines such as IL-12 [174], [175] or TLR ligands like CpG oligonucleotides [176], [177]. The latter has offered an opportunity to design a drug consisting of CpG oligonucleotides conjugated to STAT3 specific small interfering RNA (referred to as CpG-siSTAT3 conjugates) [178–180]. It has been shown in mouse cancer models and using STAT3^+^ PMN-MDSCs of prostate cancer patients that CpG-siSTAT3 conjugates mediate selective delivery of silencing siSTAT3 to TLR9^+^ myeloid cells, resulting in disruption of the STAT3 supported suppressive signaling network and stimulation of anti-tumor immunity. These findings indicate that this gene- and cell type-specific inhibitory oligonucleotides represent a novel therapeutic approach to mitigate immunosuppression in cancer patients.

## IN VITRO GENERATED MYELOID-DERIVED SUPPRESSOR CELLS: A PLATFORM FOR DRUG SCREENING

The ample evidence on the importance of STAT3 in MDSCs has instigated research into existing and novel STAT3 targeting drugs and their effect on MDSC viability and functionality (Table 1). These studies mostly use mouse MDSCs, as our knowledge of the murine system is way ahead of their human counterparts. However, studying murine MDSCs poses the technical challenge of obtaining sufficient numbers of cells at high purity from a limited number of tumors. Therefore, MDSCs from the spleen are often used as an alternative. However, spleen MDSCs are phenotypically and functionally different from tumor MDSCs [55], [181]. Consequently, to ensure reliability and potency of novel drugs, they should be evaluated on tumor rather than spleen MDSCs. To circumvent this conundrum, researchers have evaluated various *in vitro* culture systems to obtain MDSCs. These range from the use of cell lines to the differentiation of bone marrow cells [77], [80], [100], [101], [181–194]. In particular, *ex vivo* differentiation of bone marrow cells using conditioned media from GM-CSF secreting tumor cells has proven to be a successful approach [80], [175], [181], [184]. A proof-of-concept on the value of this strategy to obtain large amounts of MDSCs that resemble those found within various cancer types, including multiple myeloma, melanoma and colorectal cancer was delivered [80], [175], [181]. Since 50 to 60 million MDSCs are obtained from a single mouse without the necessity of inducing cancer, this system allows systematic and high throughput *in vitro* testing of anti-neoplastic treatments. Furthermore, these *in vitro* MDSCs offer the possibility to study the role of individual factors, including STAT3 on MDSC development [80].

Efficient *in vitro* systems should also be developed to generate human MDSCs, as this will surely facilitate research into MDSCs and more specifically STAT3-targeting drugs. Major advances have been made towards this goal. Human MDSCs have been differentiated from bone marrow cells or peripheral blood mononuclear cells using recombinant cytokine cocktails [77], [188], [189], or tumor-conditioned media [80], [101]. However, most human MDSC differentiation systems are still poorly efficient, most likely because they do not make use of fully pluripotent hematopoietic precursors [195]. Moreover, the endeavor of generating human MDSCs *in vitro* is further hampered by the lack of an in depth understanding on the nature of human MDSCs *in vivo*. Nonetheless, the study by Chen et al [190] in which GM-CSF and IL-6 were used to differentiate human MDSC from peripheral blood mononuclear cells, shows the applicability of these cells, as they possess hallmark immunosuppressive pathways, including STAT3 signaling.

## CONCLUSION

Accumulation of MDSCs in cancer patients has been linked to cancer stage and overall survival in patients with a variety of different cancer types. Therefore a strategy that targets MDSCs could be used in combination with other immune stimulatory therapies. However, due to the heterogeneity of MDSCs, both in phenotype and function, it's a challenge to pinpoint the most effective MDSC target. A large body of evidence links STAT3 activation to MDSC accumulation, differentiation and function, both direct and indirect. Because of the numerous physiological processes and signal transduction pathways that are affected by STAT3, it's likely that this can account for therapy resistance. We believe that specific STAT3 targeting in MDSCs offers great opportunities. This view has only gained in strength as increasing numbers of drugs that counteract JAK/STAT signaling have been tested in clinical trials and even have been approved by the FDA. In future multimodal treatment, specific targeting of STAT3 in MDSCs can be the way forward. This is underscored by the data provided by the CpG-siSTAT3 conjugate studies, which show that targeted delivery of STAT3 inhibiting molecules is a successful approach [177–180]. Such an approach could encompass the use of myeloid cell-targeted lentiviral vectors [196], [197] or nanoparticles [198], which could than deliver silencing RNA for STAT3 [13], [178–180] or genes encoding negative regulators of STAT3 [8–11]. Evaluating such strategies will be greatly facilitated by the use of in vitro mouse and/or human MDSCs. Extensive in vivo testing of STAT3 targeting drugs will also be required in order to determine their safety. In fact, STAT3 expressed in MDSCs is involved in homeostasis, for instance in the gut. Consequently, systemic downregulation of STAT3 in MDSCs withholds the risk of inducing immune-related adverse events such as colitis. Various studies show that myeloid cell-specific STAT3 deficiency results in enhanced inflammation and chronic colitis as a consequence of a decrease in PMN-MDSCs and linked herewith a decrease in suppressive cytokines (e.g. IL-10), an increase in pro-inflammatory cytokines (e.g. IFN-γ) and iNOS. These studies caution against systemic, long-term STAT3 depletion and highlight the necessity to develop targeted strategies [199–201].
